# A Simple Original Method of Computing Milk Formula

**Published:** 1907-02

**Authors:** L. B. Clarke

**Affiliations:** Lecturer on Diseases of Children, Atlanta School of Medicine, Atlanta, Ga.


### *A SIMPLE ORIGINAL METHOD OF COMPUTING MILK FORMULAE.*

BY L. B. CLARKE, M. D.,
Lecturer on Diseases of Children, Atlanta School of Medicine. Atlanta, Ga.

---

Necessity is the mother of invention. This axiom is as constantly applicable in medicine as elsewhere, and in the present instance was the true cause of the results now to be demonstrated.

In the writer's capacity of teacher of Diseases of Children at the Atlanta School of Medicine, necessity demanded that the student be taught some plan or scheme by which in the section devoted to the feeding of children artificially, the quantities used in milk formulæ could be readily, easily, simply and as accurately transformed into percentages, and desired percentages as easily transformed into the necessary quantity to be used in any size mixture.

This was to be accomplished in a manner that would serve to do away with cumbersome tables; with the need for memorizing a number of tabulated equivalents, and replace all of this with only a few basic principles upon which to construct the entire system.

To Dr. Emmet Holt, of New York, is due the great credit of having been the ioneer in this work, in his successful efforts to bring order out of chaos that previously existed upon this subject, resulting in his presenting to the profession certain well-established rules for feeding, tabulated accurately; the tables showing the actual strength of each ingredient entering into the mixture, i. e., fats, proteid, sugar, etc. But unless one memorizes these quantities, or carries about with him these tables written out in full a more or less intricate algebraical, or at best arithmetical problem confronts him each time he desires to compute a certain formula.

By the plan now presented, this is all obviated, and I know of no method of estimation hitherto published that is so concise and simple. Its use renders it unnecessary to remember that a given number of ounces of cream and of milk and of water make a mixture of certain percentages of fat and proteids, and unnecessary to refer to the textbook tables for the information. It is easily worked out.

The only requisite tax imposed upon memory is that which every practitioner who works at all in this line should know, viz: the three periods of infancy as applied to feeding:—

1. The early months—birth to 3 or 4 months.
2. The middle period—3 or 4 months to 9 or 10 months.
3. The later period—10 months to the end.

and that the desirable ratio of fat to proteids during these periods is as follows:

1st—3 to 1, represented in a 10% top milk basis.

2d —2 to 1, represented in a 7% top milk basis.

3d —Nearly equal, represented in a 4% or whole milk.

The method follows:

1. The fat value in any size mixture of any per cent. top milk is obtained by multiplying the number of ounces of said top milk used, by a fraction the numerator of which shall be the top milk strength, and the denominator the amount of the mixture.

The proteid value is then easily determined by its relation to the fat.

2. The number of ounces of any top milk necessary to use in any size mixture in order to produce a desired fat per cent., is obtained by reversing the above rule, i. e., dividing the known fat per cent. by a fraction obtained in the same manner as above.

The method is freely demonstrated in the following table:

| Remove Fat % | Proteid % | Ratio | | Fraction used for Fat % in any mixture | | | Proteid ¼ |
|---|---|---|---|---|---|---|---|
| Top 6th=18% | $\frac{1}{6}$ of Fat | 6–1 | $\frac{18}{2}$ | times ots. used, i. e. No. ots. used | | X $\frac{81}{\text{amt. mixt.}}$ | $\frac{6}{1}$ of Fat |
| " 5th=16% | $\frac{1}{5}$ " | 5–1 | $\frac{16}{2}$ | " | " | X $\frac{16}{\text{amt. mixt}}$ | $\frac{1}{5}$ " |
| " 4th=16% | $\frac{1}{5}$ " | 4–1 | $\frac{15}{2}$ | " | " | X $\frac{13}{\text{amt. mixt}}$ | ¼ " |
| " 3d = 10% | $\frac{1}{3}$ " | 3–1 | $\frac{10}{2}$ | " | " | X $\frac{10}{\text{amt. mixt}}$ | $\frac{1}{3}$ " |
| " ½ = 7% | $\frac{1}{2}$ " | 2–1 | $\frac{7}{2}$ | " | " | X $\frac{7}{\text{amt. mixt}}$ | $\frac{1}{2}$ " |
| Whole Milk= 4% | $\frac{7}{8}$ " | 8–7 | $\frac{4}{2}$ | " | " | X $\frac{4}{\text{amt. mixt}}$ | $\frac{7}{8}$ " |

The three last 10 per cent. 7 per cent., 4 per cent., represent the ordinary working strengths of top milk generally used in fairly normal children. Should it prove necessary to compute for a higher fat and lower proteid combination, the upper tables may be utilized.

The simplicity and practicability of the method may be demonstrated in a question on each number:

2. A. What is the fat value (i. e. Fat per cent.) in 6 oz. of 10 per cent. milk in a 24 oz mixture?

B. What is the proteid value?

Ans. A.–6 X $\frac{19}{24} = \frac{10}{4} = 2\frac{1}{2}$ per cent. Fat.

B. $\frac{1}{3}$ of the Fat—$\frac{2\text{-}2}{\text{-}2} = \frac{5}{2} \div \frac{3}{1} = \frac{5}{2} \times \frac{1}{3} = \frac{5}{6}$ per cent. proteids.

2. How much 7 per cent milk must be used in a 30 oz. mixture in order to obtain a 2 per cent. Fat?

